# An Isolated Perforation of the Fourth Part of the Duodenum Following Blunt Abdominal Trauma: A Case Report

**DOI:** 10.7759/cureus.33571

**Published:** 2023-01-09

**Authors:** Yashjot Kaur, Ravneet Kaur, Harsimrat Singh, Arpan Josan

**Affiliations:** 1 Surgery, Government Medical College, Amritsar, IND

**Keywords:** trauma, duodenal perforation, graham patch repair, enteral feeding, surgery, perforation, case report

## Abstract

An isolated perforation of the duodenum is rare in cases of blunt abdominal trauma, and diagnosis is often delayed due to subtle clinical signs. We present the case of a 13-year-old male patient who presented to the hospital with an alleged history of being run over in the abdomen by a vehicle and a complaint of severe abdominal pain. Radiography of the abdomen in the standing position showed air under the diaphragm, and ultrasonography revealed free fluid in the pelvic and peritoneal cavities, clinching the diagnosis of hollow viscus perforation. The patient was resuscitated and underwent an exploratory laparotomy under general anesthesia. On exploration, no perforation was found in the intraperitoneal organs. The retroperitoneum was opened, and the Cattell-Braasch maneuver was used to approach the duodenum. A single perforation was discovered in the fourth part, and a modified graham patch repair was done. As soon as the patient's bowel sounds returned, a low-fat diet was started through a nasojejunal tube placed distal to the perforation site. The patient was discharged in good condition and is doing well with regular follow-ups. This case emphasizes the need for a high index of suspicion for gut perforation of both intra- and retroperitoneal organs after blunt trauma. This will help in early diagnosis and timely management to reduce perforation-associated mortality. Damage control surgery, with primary closure of the perforation, is well-suited and preferred in an emergency and resource-limited setting.

## Introduction

Duodenal perforation as a result of blunt abdominal trauma is an extremely rare condition that occurs in only 2% to 10% of cases. It is most common in children due to their weak muscles and thin abdominal wall [1]. Except for the duodenal bulb, the first, second, third, and fourth parts of the duodenum are retroperitoneal in location [2]. The fourth segment is 2.5 cm long and runs up and to the left side of the aorta, in front of the second lumbar vertebra, to join the jejunum at the duodenojejunal flexure. The duodenum is fixed to the diaphragm with the ligament of Treitz (at the duodenojejunal flexure). This increases the risk of perforation during blunt abdominal trauma due to compression of the duodenum against the lumbar vertebrae [3]. The patient may present with mild abdominal pain. Perforation peritonitis caused by the irritating contents of the duodenal juices, particularly bile, can present as an abdominal emergency, with mortality rates as high as 19% [4]. Abdominal X-ray may show air under the diaphragm or retroperitoneal air bubbles. An abdomino-thoracic contrast-enhanced computed tomography (CECT) scan is the gold standard for diagnosis [5] and may show pneumoperitoneum, retroperitoneal air, paraduodenal collection of fluid, and extravasation of the contrast. Because diagnosis is often difficult [6], and even delayed, one must maintain a high index of suspicion for duodenal perforation in children sustaining blunt injuries to the abdomen. This report presents the case of a 13-year-old boy who presented to the hospital with a history of blunt abdominal trauma from a road traffic accident, complaining of severe abdominal pain.

## Case presentation

A 13-year-old male was involved in a run-over road traffic accident with a light vehicle. He was taken to the peripheral primary care hospital in severe pain but vitally stable. First aid and pain medication were provided but had minimal improvement. Hence, he was referred to the tertiary care hospital. On arrival, the patient was quickly evaluated based on the advanced trauma life support (ATLS) protocols. He was normotensive (blood pressure 106/68 mmHg), but his heart rate was high (126 beats per minute). There were no external signs, including bruises. On chest auscultation, there was bilateral equal air entry with bibasilar crepitation. The abdomen was tense, distended, and tender with active guarding and rigidity. Bowel sounds were absent on auscultation of the abdomen.

**Figure 1:**
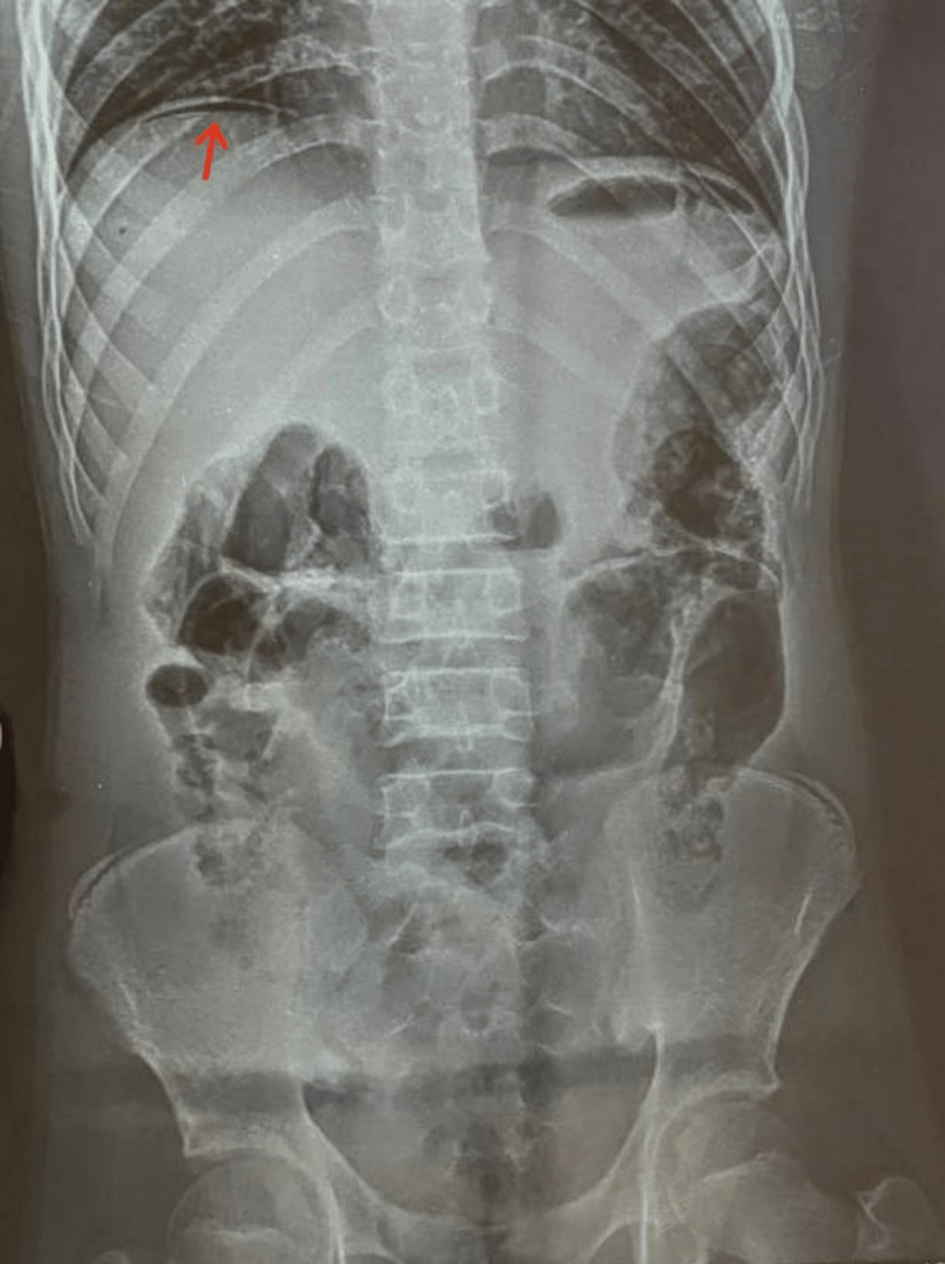
Radiograph of the abdomen in the standing position showing air under the right hemidiaphragm, as indicated by the arrow.

X-ray of the abdomen in the standing position showed air under the right hemidiaphragm, suggestive of hollow viscus perforation (Figure 1). Ultrasonography of the abdomen revealed mild intraperitoneal free fluid. It was also suggestive of a small left renal contusion and hemorrhagic cystitis. Due to the emergent nature of the case, CT scans were not ordered and the patient was shifted to the intensive care unit. Blood investigations revealed a total leucocyte count of around 2,300 mm^-3^ (reference range 3,500-9,500 mm^-3^), elevated urea, and creatinine of values 68.0 mg% (<45 mg%) and 1.65 mg% (<1.5 mg%), respectively. Liver enzymes - serum glutamic oxaloacetic transaminase (SGOT), serum glutamic pyruvic transaminase (SGPT), and serum alkaline phosphatase (ALP) were elevated and measured 88 IU/L (0-40 IU/L), 57 IU/L (0-40 IU/L), and 154 IU/L (25-150 IU/L), respectively. Both the prothrombin index (PTI) and the international normalized ratio (INR) were within normal limits.

**Figure 2:**
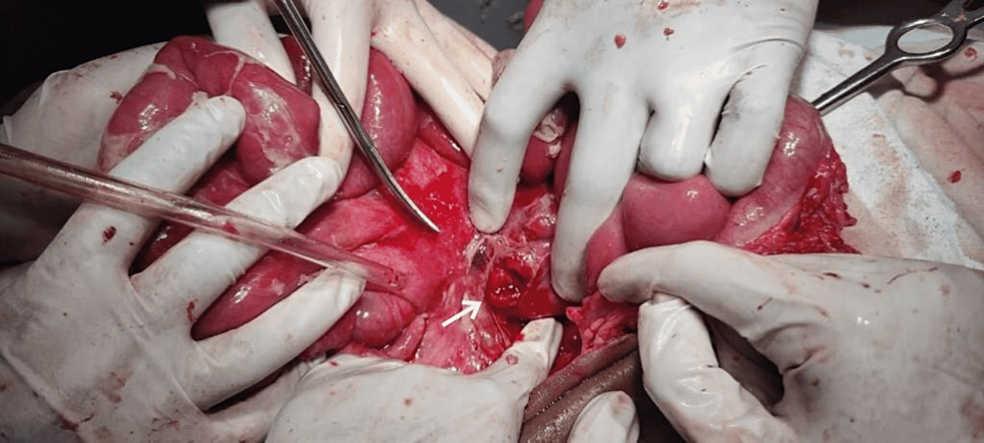
Intraoperative image showing the perforation of the fourth part of the duodenum, as depicted by the arrow.

**Figure 3:**
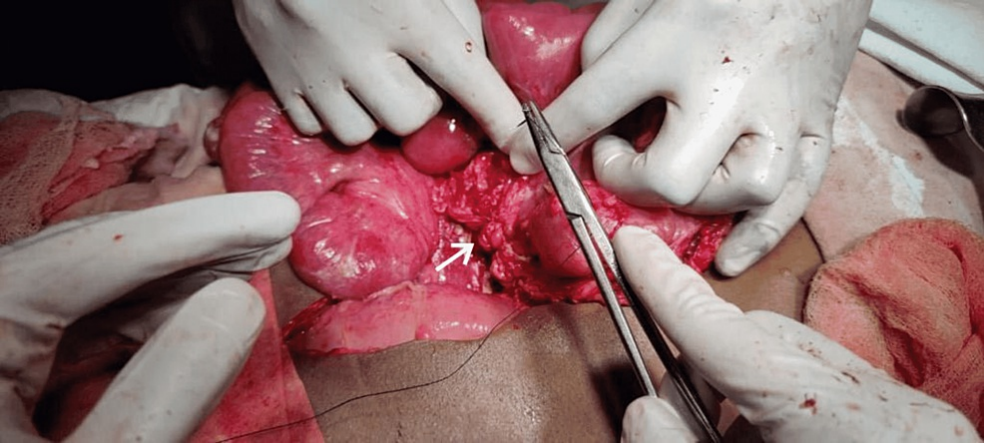
Intraoperative image showing the closure of the defect using the omental patch, that is, modified Graham patch repair, as depicted by the arrow.

Based on presentation and imaging, the patient was diagnosed with perforation peritonitis. He was immediately shifted to the intensive care unit, resuscitated, and prepared for emergency laparotomy. The patient underwent exploratory laparotomy under general anesthesia. Free fluid was seen in the peritoneum. However, no perforation was found in the intraperitoneal viscera. There was no evidence of solid organ injury either. Using the Cattell-Braasch maneuver, the retroperitoneal space was opened. A small (1.2 cm × 1.3 cm) single perforation was found on the anterior wall of the fourth part of the duodenum (Figure 2). The rest of the duodenum was intact. The perforation was repaired with a Graham patch omentopexy (Figure 3), and a nasojejunal tube was placed beyond the site of the perforation. 

The patient received intensive care for five days after surgery. The patient was kept nil per oral for four days postoperatively and was given parenteral nutrition. Enteral feeding with a low-fat diet was started on the fifth day through the nasojejunal feeding tube. The patient passed stool on the seventh day. Oral feeds were also started on the seventh day. After successful recovery, the patient was discharged on day 21. The patient did not encounter any complications during the hospital stay. He was admitted for this extended period for nutritional rehabilitation because the family being migratory workers did not have access to good-quality nutrition. He was followed up after six months with a CT scan, which was unremarkable.

## Discussion

Duodenal perforation as a result of blunt abdominal trauma is an extremely rare condition that occurs in only 2% to 10% of cases, and it is most common in children due to their weak muscles and thin abdominal wall [1]. Duodenal perforation has many causes, including peptic ulcer disease, iatrogenic causes such as post-endoscopic retrograde cholangiopancreatography (ERCP), and trauma. In this case report, we focus on duodenal perforation secondary to blunt abdominal trauma. The patient may initially complain of slight abdominal pain. Perforation peritonitis caused by the irritating contents of the duodenum, particularly bile, can present as an abdominal emergency, with mortality rates as high as 19% [4]. It needs to be treated meticulously as the diagnosis may be delayed due to its subtle clinical and radiological findings [7]. Shock is one of the major causes of preventable mortality in these patients. Therefore, preoperative resuscitation in the form of aggressive fluid therapy is essential for decreasing mortality associated with trauma-related perforations [8]. Emergent exploratory laparotomy is a lifesaving procedure in duodenal perforation. The aim is to perform a damage control surgery, with primary closure being adequate for isolated perforations [9,10].

A vast array of surgical options are available, ranging from minimal to extensive procedures. The most commonly employed and easiest procedure with good outcomes is primary repair using the pedicled omental patch, also known as the Graham patch repair [11,12]. The other procedure performed is triple tube drainage (TTD), consisting of tube gastrostomy, retrograde tube duodenotomy, and feeding jejunostomy, which helps in the decompression of the duodenum [13]. In an emergency setting, as in the case presented, the patient cannot bear a huge surgical intervention, and due to the inherent economic constraints, we preferred to do a modified Graham patch repair.

Starting enteral feeding, as early as within the first 24 hours - early enteral nutrition (EEN), has been found to be safe with reduced postoperative complications [14]. In our case, we started the enteral feeds on the fifth day - the day bowel sounds returned. Duodenal fistula is a dreaded complication of the primary repair [15], due to the irritable duodenal contents, mainly the bile, which contains bile acids. We attempted to deal with this in our case by starting enteral feeding via the nasojejunal tube with a low-fat diet, which decreases bile secretion [16], thus decreasing the possibility of the formation of a fistula. Moreover, aspiration of bile and duodenal contents was done from time to time from the nasojejunal tube to decrease the exposure of the repair site to the bile.

## Conclusions

Duodenal perforation is rare in blunt trauma patients. It may present with subtle clinical signs, which may delay the diagnosis. Therefore, one must keep a high index of suspicion for duodenal perforation in children presenting with blunt abdominal trauma, especially in cases when no perforation is discovered in the intraperitoneal organs during an exploratory laparotomy. Performing damage control surgery with minimal procedures is ideal in a case presenting as an emergency, especially in critically ill patients.
